# Advances in Plant Regeneration: Shake, Rattle and Roll

**DOI:** 10.3390/plants9070897

**Published:** 2020-07-16

**Authors:** Sergio Ibáñez, Elena Carneros, Pilar S. Testillano, José Manuel Pérez-Pérez

**Affiliations:** 1 Instituto de Bioingeniería, Universidad Miguel Hernández, 03202 Elche, Spain; s.ibanez@umh.es; 2 Pollen Biotechnology of Crop Plants Group, Margarita Salas Center of Biological Research, CIB Margarita Salas-CSIC, Ramiro de Maeztu 9, 28040 Madrid, Spain; ecarneros@cib.csic.es (E.C.); testillano@cib.csic.es (P.S.T.)

**Keywords:** hormone-induced callus, wound-induced callus, somatic embryogenesis, stress-induced microspore embryogenesis, root tip regeneration

## Abstract

Some plant cells are able to rebuild new organs after tissue damage or in response to definite stress treatments and/or exogenous hormone applications. Whole plants can develop through de novo organogenesis or somatic embryogenesis. Recent findings have enlarged our understanding of the molecular and cellular mechanisms required for tissue reprogramming during plant regeneration. Genetic analyses also suggest the key role of epigenetic regulation during de novo plant organogenesis. A deeper understanding of plant regeneration might help us to enhance tissue culture optimization, with multiple applications in plant micropropagation and green biotechnology. In this review, we will provide additional insights into the physiological and molecular framework of plant regeneration, including both direct and indirect de novo organ formation and somatic embryogenesis, and we will discuss the key role of intrinsic and extrinsic constraints for cell reprogramming during plant regeneration.

## 1. Introduction

Unlike what happens in animals, plants have a high regenerative capacity and, under natural conditions, they are able to form new organs and even complete individuals from a few cells present in adult tissues, either in response to injury or to the alteration of their environment [1]. Classical in vitro culture experiments of plant tissues indicated that the exogenous auxin and cytokinin (CK) balance control plant organogenesis, so that a high CK-to-auxin balance induces the production of shoots, an elevated auxin-to-CK balance induces the formation of roots, while intermediate levels of both hormones induces the formation of an amorphous cell mass dubbed callus [2]. Application of stress treatment or exogenous auxin can induce somatic embryogenesis, an intriguing process that exemplifies plant cell totipotency expression. Here, we provide an update on the key molecular and signaling events on three different regenerative processes in plants: (i) hormone-induced callus formation; (ii) tissue regeneration after micro-surgical excision of the root tip; and (iii) embryo induction in somatic cells from different cell types and explants without the fusion of gametes.

## 2. Transcription Factor Networks and Epigenetic Regulators during Hormone-Induced Callus Formation

**Figure 1:**
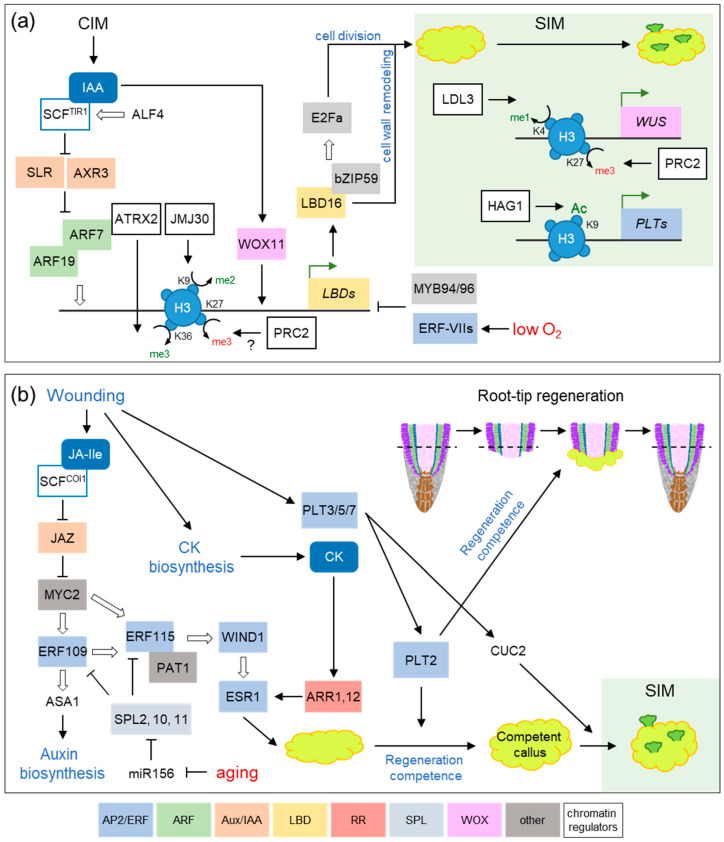
**Transcriptional networks involved in cell reprogramming during regeneration.** (**a**) Hormone-induced shoot organogenesis. (**b**) De novo root formation after root-tip excision. Positive (wounding, callus-inducing medium (CIM), auxin, etc.) and negative (low O_2_, aging, etc.) signals are shown in blue and red, respectively. Transcriptional and epigenetic regulators (Table 1) are depicted inside boxes of different colors. Each color represents a given DNA binding domain (see main text for legends). White arrows indicate direct upregulation via promoter binding.

Callus formation is experimentally induced from a variety of plant tissues by their incubation on an auxin-rich callus-inducing medium (CIM), and relies on the re-deployment of lateral root (LR) developmental programs from existing pericycle-like cells (Figure 1a), which are functionally analogous to animal stem cells [3]. Hence, mutants defective in LR formation, such as *aberrant lateral root formation 4* (*alf4*) and *solitary root 1* (*slr1*, also known as *iaa14*) are also impaired in auxin-induced callus formation [3]. ALF4 is required for the formative divisions of xylem pole pericycle (XPP) cells during LR formation [4]. Through its binding to the RBX1 subunit of the SCF E3 ligases, ALF4 interferes with the interaction between E2 and RBX1 [5]. As a result, several SCF^TIR1^ substrates, such as AUXIN RESISTANT 3 (AXR3), are miss-regulated in the *alf4* mutants and this might explain the auxin-related phenotypes of *alf4* seedlings [5]. AXR3 physically interacts with MONOPTEROS, also known as AUXIN RESPONSE FACTOR 5 (ARF5), and the resulting AXR3-ARF5 complex functions as a transcriptional repressor at low auxin levels [6], which has also been shown to control plant stem cell maintenance and differentiation during embryogenesis [7].

Several LATERAL ORGAN BOUNDARIES DOMAIN (LBD) transcription factors (LBD16, LBD17, LBD18 and LBD29) act downstream of the auxin-responsive transcription factors ARF7 and ARF19 to induce callus formation, to some extent through regulation of the E2 PROMOTER BINDING FACTOR a (E2Fa) transcription factor that promotes cell division [8]. Accordingly, the *arf7 arf19* double mutants display reduction of auxin-induced callus formation [9], while the ectopic expression of each of these LBD genes is sufficient to trigger callus formation in the absence of exogenously applied auxin [8]. In addition, auxin activates the expression of *WUSCHEL RELATED HOMEOBOX 11* (*WOX11*) and its homolog *WOX12*, which in turn have been shown to induce *LBD16* and *LBD29* expression during hormone-induced callus formation [10].

The chromatin context influences the accessibility of transcriptional regulators and thereby gene expression profiling during cell reprogramming and regeneration (Figure 1a) [11,12,13]. The ARABIDOPSIS TRITHORAX-RELATED 2 (ATXR2) is a histone lysine methyltransferase that stimulates the deposition of the active H3K36me3 mark at the *LBD16* and *LBD29* promoters through its direct interaction with ARF7 and ARF19 transcription factors [9]. Hence, ATRX2 contributes to the auxin-mediated epigenetic regulation of *LBD* expression during callus formation (Figure 1a) [9]. JUMONJI C DOMAIN-CONTAINING 30 (JMJ30), also known as JMJD5, is a member of the JmJC domain subgroup of histone demethylases that is involved in diverse developmental processes, including circadian regulation and temperature-dependent flowering control [14,15]. JMJ30 is recruited to the promoters of the *LBD16* and *LBD29* genes by ARF7 and ARF19, and removes the repressive H3K9me3 mark to ensure chromatin-dependent activation of *LBD* expression during hormone-induced callus formation [16]. Moreover, the ARF-JMJ30 complex further recruits ATXR2, and the multimeric protein complex ensures stable *LBD* activation during callus formation [16].

Di- and tri-methylation of Lys27 on histone H3 (H3K27me2/3), catalyzed by the Polycomb repressive complex 2 (PRC2), is a key repressive mark of many developmental processes in eukaryotes [17]. Earlier work suggested a central role of H3K27me3 mark during plant regeneration, particularly on genes on the auxin biosynthesis and root development pathways, where the H3K27me3 levels decreased during callus formation [18]. In leaf explants, the early activation of the auxin biosynthesis genes *YUCCA1* (*YUC1*) and *YUC4* during de novo root regeneration is accompanied by decreasing H3K27me3 levels at their promoters [19]. The high CK levels of the shoot-inducing medium (SIM) gradually reduced H3K27me3 levels at the *WUSCHEL* (*WUS*) locus in a cell cycle-dependent manner allowing its expression and an efficient shoot regeneration [20]. Callus-promoting LBDs are known H3K27me3 target genes [21] and it would be interesting to test whether removal of this repressive histone mark in the *LBD* promoters is required for auxin-induced callus formation.

LYSINE-SPECIFIC DEMETHYLASE 1, LSD1 (also known as KDM1A), is a conserved histone demethylase in metazoans that specifically removes H3K4me1/me2 or H3K9me1/me2 marks, and can function as a transcriptional repressor or activator [22]. The Arabidopsis genome contains four *LSD1* paralogs, *FLOWERING LOCUS D* (*FLD*), *LDL1*, *LDL2* and *LDL3*, which have been linked to seed dormancy, circadian clock and flowering time regulation [23,24,25]. In a recent report, *LDL3* was found upregulated on CIM and presumably removes H3K4me2 during callus formation, which then may allow the genes for shoot initiation to be expressed after SIM treatment [26]. In human cells, LSD1 participates in the maintenance of stem cell pluripotency through the control of the levels of H3K4 methylation at the regulatory regions of some Oct4-regulated developmental genes involved in the cellular balance between self-renewal and differentiation [27]. One possible scenario for LDL3 function is that stepwise histone modifications take place between the LDL3-mediated primed H3K4me2 demethylation in CIM treatment and the gene activation in the subsequent SIM treatment [26]. 

The Arabidopsis histone acetyltransferase HAG1, also known as GENERAL CONTROL NONREPRESSED 5 (GCN5), was previously reported to affect the stem cell niche maintenance in roots by regulating *PLETHORA1* (*PLT1*) and *PLT2* expression [28]. HAG1 plays a pivotal role in the establishment of pluripotency in callus and subsequent shoot regeneration [29]. In developing CIM-induced callus, HAG1 catalyzes histone acetylation at several root-meristem loci, including *PLT1*, *PLT2*, *SCARECROW* (*SCR*) and *WOX5*, which drives their transcriptional activation allowing successful shoot regeneration after incubation on SIM [29]. In human gastrointestinal endocrine cells, LSD1-mediated H3K9me2 demethylation facilitate subsequent histone H3K9 acetylation by histone acetyltransferases, leading to gene activation [30]. Likewise, in Arabidopsis shoot regeneration, HAG1 might play roles in the LDL3-mediated gene priming, a hypothesis that now might be tested.

In a search for additional regulators of hormone-induced callus formation, the BASIC REGION/LEUCINE ZIPPER MOTIF 59 (bZIP59) transcription factor was identified on a screen for LBD17-partners, and its physical interactions with the other LBDs involved in auxin-induced callus formation were confirmed [31]. Interestingly, CIM or auxin treatment induced a post-translational accumulation of bZIP59 specifically in pericycle-like cells, and that enhanced its interaction with LBD16. Further results confirmed that bZIP59 and LBD16 act synergistically on a subset of LBD target genes that might directly contribute to callus formation [31]. Among the upregulated LBD targets identified so far [32], genes involved in cellular oxygen availability and activation of reactive oxygen species (ROS), cell wall remodeling and lipid metabolism deserve further investigation.

Additional regulation of callus-promoting LBD function by two MYB-domain transcription factors, have been recently described [33]. MYB94 and MYB96 regulate *LBD29* expression during callus formation through direct binding to its promoter, likely through the inhibition of pericycle-like cell competence in a novel, unknown regulatory pathway [33]. These MYB-domain transcription factors are involved in lipid metabolism in response to ABA or abiotic stress (i.e., drought and cold) by regulating the biosynthesis of very-long-chain fatty acids (VLCFAs). In plants, VLCFAs participate in the regulation of organ regeneration processes through its negative role in pericycle-like cell competence during auxin-induced callus formation [34]. Wild-type plants treated with a VLCFA biosynthesis inhibitor and mutants with altered VLCFA biosynthesis exhibited an over proliferation of cells in the leaf vasculature, a phenotype that was dependent on endogenous CK levels [35]. VLCFAs or their derivatives act non-cell autonomously to restrict pericycle-like cell competence and thereby prevent excess callus formation in response to external cues [34]. Interestingly, plasma membranes across juxtaposed cells display enrichment in sterols and sphingolipids with saturated VLCFAs that functionally define the plasmodesmata domain [36]. A direct link between VLCFA metabolism, plasmodesmata function and cell-to-cell trafficking has been recently established between sieve elements and phloem pole pericycle cells [37]. It is tempting to speculate that analogous cell-to-cell trafficking of an unknown non-cell autonomous signal (maybe acting on ALF4 regulation) between XPP and neighboring cells might restrict pericycle cell competence during regeneration.

## 3. Wound Signaling Regulates Tissue Regeneration through Conserved Gene Regulatory Networks

Our understanding of the molecular networks involved in wound-induced tissue regeneration has gained from recent results (Table 1) [38]. In *Arabidopsis thaliana*, the micro-surgical excision of the root tip leads to a quick re-specification of lost cell identities and to the re-establishment of a functional stem cell niche that allows complete organ regeneration (Figure 1b) [39]. By a combination of lineage tracing, single-cell RNA sequencing and marker analysis, it was shown that stem cells originate de novo from multiple tissues near the wound, on a process that required the activation of the MONOPTEROS transcription factor which is normally required for hypophysis specification during the formation of the embryonic root [40]. In addition, self-organizing auxin and CK interactions near the wound reset cell identities in this region and provide new positional cues to the dividing cells of the remaining meristem for the re-establishment of the developmental axes within the newly formed tissues [40].

The AP2/ERF transcription factor WOUND INDUCED DEDIFFERENTIATION 1 (WIND1), also known as RAP2.4, was identified as a central regulator for wound-induced cellular reprogramming in plants [41]. WIND1 is sufficient to establish and maintain dedifferentiated cell status without the exogenous addition of auxin and CKs. WIND1 is induced at the wound site where it promotes cell proliferation by the direct upregulation of ENHANCER OF SHOOT REGENERATION 1 (ESR1) [42]. Based on expression data and mutant analyses, CKs activate ESR1 expression through the B-type ARABIDOPSIS RESPONSE REGULATOR 1 (ARR1) and ARR12 [41,42]. Indeed, the *arr1 arr12* double mutants displayed reduced callus formation at wounded hypocotyls after shoot excision [43] but decreased rooting capacity from leaf explants [44], suggesting a complex regulation of CK signaling during tissue regeneration.

A recent study has contributed to clarify the intriguing results found for ARR1 and ARR12 in different regeneration models. ARR12 is a central enhancer of both callus formation and shoot regeneration whereas ARR1 inhibits regeneration through transcriptional activation of *AXR3* and that indirectly repress *WUS* expression [45]. Interestingly, MONOPTEROS binds the promoter of ESR1 and directly represses its transcription, providing a mechanistic model for auxin and CK crosstalk during regeneration [7]. ETHYLENE RESPONSE FACTOR 115 (ERF115), which was initially described as a rate-limiting factor for quiescent center (QC) cell division after DNA damaging stress [46], has been found to upregulate *WIND1* expression through its heterodimerization with PHYTOCHROME A SIGNAL TRANSDUCTION 1 (PAT1) [47]. These results are in agreement with a role of ERF115-PAT1 complex in driving the regeneration potential of root meristem cells in response to local cell death caused by wounding. However, the direct link between the wound signal and WIND1 expression have remained elusive until recently. Latest studies have shown that wounding produces changes in the H3K9/14 and H3K27 acetylation state of key reprogramming genes such as *WIND1*, *ERF113* or *LBD16* [48]. Moreover, it has been described that the histone variant HISTONE THREE RELATED 15 (H3.15), which lacks the PRC2-targeted K27 residue, is quickly induced after wounding. The absence of the H3K27me3 repressive mark in the H3.15 histones causes the de-repression of several key developmental genes, amongst which is *WOX11* [49]. The repressive mark H3K27me3 seems to be conserved in regenerative processes along the plant lineage. Indeed, the ectopic expression of the AP2/ERF-encoding gene *STEM CELL-INDUCING FACTOR 1* (*STEMIN1*) in *Physcomitrella patens* leaves causes the acquisition of stem cell properties in leaf cells through local reduction of H3K27me3 marks before cell division in a subset of STEMIN1 targets [50].

The stress hormone jasmonic acid (JA) plays well-established roles in wounding and defense responses. Downstream of the JA signal, the F-box protein CORONATINE INSENSITIVE1 (COI1) binds to JA and destabilizes the JA ZIM domain (JAZ) repressor proteins, allowing the positive regulators, such as the basic helix-loop-helix (bHLH)–domain containing MYC transcription factors, to induce their target genes [51]. JA promotes de novo root formation in Arabidopsis leaf explants [52]. After leaf excision, free JA and its active form JA-isoleucine (JA-Ile) are quickly upregulated within 10 to 30 min, and a time-course RNA-seq analysis identified the *ERF109* gene as a key factor for root regeneration [52]. Additionally, ERF109 was found to directly upregulate *ANTHRANILATE SYNTHASE α1* (*ASA1*), which encodes an enzyme involved in the tryptophan biosynthesis pathway [52]. Tryptophan is the precursor of auxin which, in turn, is upstream of the WOX11 activation required for hormone-induced callus formation (see above), as well as for de novo root regeneration [53]. The role for ERF109 in tissue regeneration, downstream of MYC2, was independently confirmed using the root-tip excision model [54]. Interestingly, the levels of *ERF109* induction after root tip excision depended on the position of the cut along the proximodistal axis of the root, which may correlate with the regeneration capacity of remaining tissues and was restricted to the root-ward region of the meristem [54]. Additionally, ERF109 was found to upregulate *ERF115* expression in cooperation with unknown auxin transcriptional regulators [54].

The molecular mechanism that restricts regenerative capacity during tissue culture along with plant age has been well-documented [55]. microRNA156 (miRNA156) repress the expression of several *SQUAMOSA PROMOTER BINDING PROTEIN-LIKE* (*SPL*) genes, which causes progressive decline in shoot regeneration [56]. In addition, a role for miR156 during de novo root formation was proposed based on the reduced number of wound-induced ARs of plants transformed with *35S::MIM156*, which blocks the activity of miR156 and causes an increase in SPL expression [57,58]. In older leaves, SPL2, 10 and 11 directly bind to the promoters of a subset of wound-induced AP2/ERF transcription factors, such as ABR1, ERF109, ERF115 and RAP2.6L, among others, and attenuate their induction, thereby dampening auxin accumulation at the wound (Figure 1b) [59].

Wound stress activates a set of AP2-ERF transcriptional regulators, including WIND1, WIND3, RAP2.6L, ERF114, ERF115, PLT3, PLT5 and PLT7, and they contribute to callus formation at wound sites [55]. It was described that *PLT3*, *PLT5* and *PLT7* regulate de novo shoot formation in root and hypocotyl Arabidopsis explants under CIM and subsequent SIM culture conditions [60]. *PLT3, PLT5* and *PLT7* are upregulated in mitotically active cells of callus tissue, regardless of the explant type, and their expression is progressively confined in clusters of cells forming the new shoot primordia upon their transfer to SIM [60]. Although the incubation of *plt3 plt5-2 plt7* explants in CIM can successfully achieve the formation of a callus mass, later culture of these *plt3 plt5-2 plt7* calluses in SIM did not produce any adventitious shoots, indicating their function is not essential during the reversion of the explant identity or during callus proliferation, but required for shoot initiation [60]. The authors demonstrated that PLT5-mediated induction of *PLT2* is required for calluses to develop shoot primordia, as this root stem cell regulator confers the regeneration competence required for shoot initiation from callus tissue. LR primordia exposed to high concentrations of CKs ectopically express *PLT3, PLT5* and *PLT7*, which induce subsequent *PLT2* expression and lead to direct de novo shoot regeneration [60]. In line with the proposed role for PLT2 regarding regeneration competence acquisition during indirect adventitious shoot formation, LR primordia of *plt3 plt5-2 plt7* mutants were not able to induce *PLT2* expression and no successful direct de novo shoot regeneration process was observed [60]. In this context, it was proposed that PLT2 is also responsible for the regeneration competence in ablated or completely RAM-excised roots, which undergo root meristem regeneration. The endogenous gradient of PLT2 of undamaged root tips determines the competence for root tip regeneration, and the transient overexpression of PLT2 confers regeneration potential to differentiating cells beyond the regeneration competence region, which usually comprises the last 210-250 µm of the root meristem [61]. In addition, the reduction of retinoblastoma-related (RBR) levels enhances the effect of PLT2 overexpression and leads to the re-entry of differentiated cells into organ formation programs [62]. Interestingly, the JA-triggered activation of root stem cells through the RBR-SCR network and stress response protein ERF115 leads to the restoration of root tip lost after resection [54]. As, such that the decline of PLT2 towards the shoot-ward end of meristem is causal for the drop in regeneration capability at this region [61].

A recent paper [63] shows that PLT3, PLT5 and PLT7 promote YUC4-mediated local auxin biosynthesis to induce procambium proliferation and vascular regeneration in damaged aerial organs, although, in this process, they seem to perform through *CUP-SHAPED COTYLEDON 2* (*CUC2*) induction, instead of *PLT2* [63]. As the adventitious roots arise from cambium tissue in the majority of plant species, it would be interesting to explore whether this regeneration module is conserved in other types of regeneration processes.

## 4. Somatic Embryogenesis: Stress, Auxin and Epigenetic Modifications as Key Players of Cell Totipotency Expression

**Figure 2:**
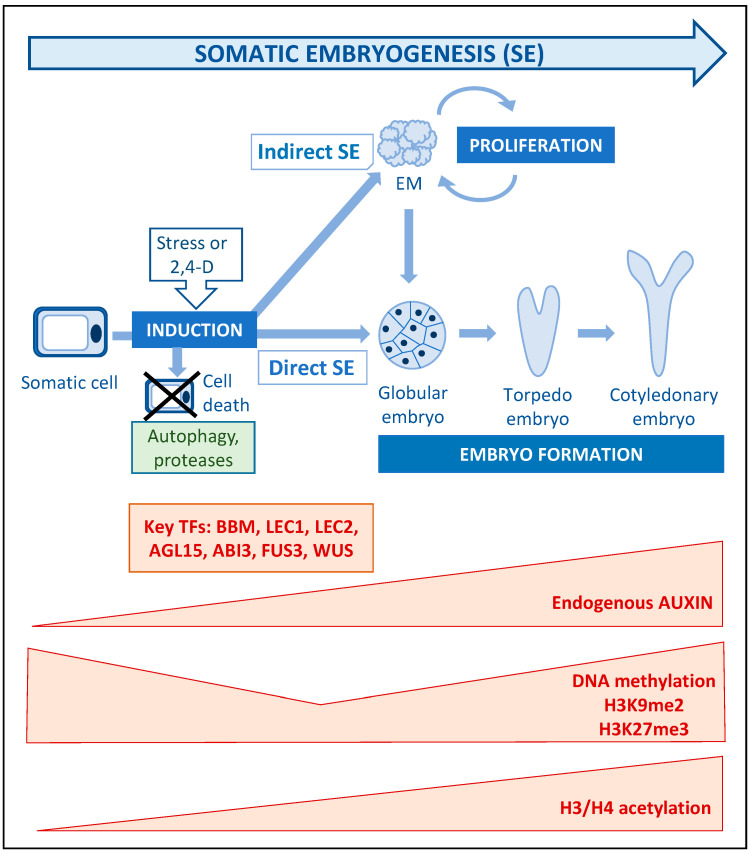
**Schematic overview of somatic embryogenesis stages along the process**. The presence and intensity of main regulatory factors (transcription factors, epigenetic modifications and auxin) are indicated in orange boxes (Table 1). Triggering factors and collateral cell death related processes are indicated in grey and green boxes, respectively. EM: embryogenic masses.

The high regeneration competence of plants derives from the extreme developmental plasticity of plant cells that allows the formation of organs and bipolar embryos under specific conditions. Somatic embryogenesis (SE), the induction of embryos from different cell types and explants, without the fusion of gametes, is one of the best examples of plant cell totipotency [64,65]. SE induction can lead to the formation of embryos directly from a cell or group of cells of the explanted tissue (direct SE), or to the proliferation of masses of embryogenic cells that further produce embryos (indirect SE) [66,67] (Figure 2). Despite this process having been extensively studied as a plant regeneration model, an understanding of the regulatory mechanisms at the molecular and cellular levels is still elusive.

SE is considered a very powerful tool in plant biotechnology, as a feasible in vitro procedure for plant cloning and regeneration purposes [64]. Due to its great potential for large-scale clonal propagation and the cryopreservation of elite genotypes, as well as for production of genetically modified plants with improved traits, SE has been proven to be very useful for propagation of species with long reproductive cycles or low seed set in a large variety of crop and forest species [64,68,69,70]. In the case of microspore embryogenesis, the microspore (haploid cell, precursor of pollen grain) is reprogrammed towards an embryogenic pathway, by stress treatment [71]. The resulting haploid embryo, after spontaneous or chemically-induced diploidization, will produce doubled-haploid plants [72,73,74], which are widely used by seed and horticulture companies, since they provide unique source of new genetic variability, are homozygous at all genomic loci, and the allele fixation is accomplished very quickly, as compared to assortative mating schemes, like self-pollination [75,76]. Although SE is currently widely exploited, it is still highly, or even completely, inefficient in many species of economic interest. The low efficiency of embryo production in recalcitrant species presents serious limitations for widespread application of SE in the fields of agriculture and forestry. Together with its biotechnological application, SE represents a very interesting model to study cell reprogramming, totipotency acquisition and embryogenic development, processes that involve the action of a complex signaling network which is not well understood yet.

The induction of SE is a multi-factorial developmental process that is usually initiated in response to exogenous stimuli produced by hormones, certain stress treatments (low or high temperature, osmotic shock, drought), or by a combination of both types of inductive conditions [65,66]. The stress treatment applied to switch the cell developmental program can also produce cell damage, and even partial or complete cell death. Recent reports have indicated that stress-induced cell death is a major factor that greatly reduces the yield of SE in various in vitro systems, particularly in microspore embryogenesis [77,78]. Markers of cellular death such as autophagy, the major catabolic process of eukaryotic cells, and cell death proteases (metacaspases, cathepsins and proteases with caspase 3-like activity) are activated during stress-induced microspore embryogenesis [71,79,80,81,82]. Pharmacological treatments with inhibitors of autophagy and proteolytic activities lead to the reduction of cell death, consequently increasing the embryogenesis initiation rate [79,80,81]. These novel findings are paving the way for new intervention pathways to increase cell viability in SE cultures.

The progress obtained on somatic embryogenesis in Arabidopsis has allowed the characterization of some genes involved in the molecular mechanisms underlying the complex regulatory networks that control SE. Exogenous auxins, either alone or in combination with other plant growth regulators, or stress, induce SE and the expression of different genes. Key transcription factors that have been found upregulated during the induction of SE in different species are members of the AINTEGUMENTA-LIKE (AIL) family, like BABY BOOM (BBM), PLT1 and PLT2, and others, such as AGAMOUS LIKE 15 (AGL15), FUSCA 3 (FUS3), LEAFY COTYLEDON 1 and 2 (LEC1, LEC2), RWP-RK DOMAIN-CONTAINING 4 (RKD4), ABA INSENSITIVE 3 (ABI3), and WUSCHEL (WUS) [66,83,84] (Table 1 and Figure 2). Some of these genes, such as *WUS*, *LEC1*, *LEC2* or *BBM*, have been reported to be responsible for the meristem/embryo identity during normal development, and their ectopic expression can also directly reprogram somatic cells and induce SE in the absence of exogenous stimuli [83].

The evidence supports the notion that auxins play a critical role in the reprogramming of somatic cells to SE [69,71]. In many SE protocols, treatment with exogenous auxin (usually 2,4-dichlorophenoxyacetic acid, 2,4-D) results in cell reprogramming, while SE initiation requires the subsequent elimination of auxin from culture media [65]. It has been proposed that the addition of 2,4-D to the culture medium induces an embryogenic response that is associated with the increase of the endogenous levels of indole-3-acetic acid (IAA) [84]. In various species, endogenous IAA levels have been shown to increase during SE initiation and embryo development [66,85,86]. In the microspore embryogenesis of *Brassica napus* and *Hordeum vulgare*, cell reprogramming is induced by stress without exogenous auxin in the culture media. However, endogenous auxin levels are highly increased in these species from the first embryogenic divisions in 2-3 cell proembryos [85,87] (Figure 2). Furthermore, SE is accompanied by the activation of endogenous auxin biosynthesis, polar transport and signaling pathways, as indicated by the up-regulation of auxin biosynthesis genes *YUC*, *TRYPTOPHAN AMINOTRANSFERASE OF ARABIDOPSIS 1* (*TAA1*), and *TRYPTOPHAN AMINOTRANSFERASE-RELATED 2* (*TAR2*), polar transport gene *PIN-FORMED 1* (*PIN1*) and signaling genes *Aux/IAA* and *ARF* [85,86,87,88,89]. Interestingly, the use of inhibitors of auxin biosynthesis (kynurenine), polar auxin transport (N-1-naphthylphthalamic acid), and auxin antagonists (α-(pchlorophenoxy)-isobutyric acid), drastically impairs SE in monocot and eudicot species [85,87], indicating the key role played by this hormone in the process.

Together with hormones, epigenetic marks play an important role during SE induction and progression. Chromatin-modifying factors regulate conformational states of chromatin and its accessibility to transcriptional machinery. Epigenetic modifications, mainly DNA methylation and histone methylation and acetylation, are key factors contributing to the functional status of chromatin, that regulates gene expression, during cell proliferation and differentiation in both animals and plants [90]. In SE studies, the totipotency of cells was found to be associated with an open chromatin conformation [91]. Many studies have reported the ubiquitous epigenetic changes associated with SE initiation. In particular, it has been found in a number of species that initial stages of cell reprogramming and embryogenesis initiation usually involve widespread DNA hypomethylation [91,92,93,94], histone H3K9 demethylation [95,96,97] and histones H3 and H4 acetylation [83,96] (Figure 2). In Arabidopsis, H3K27 methyltransferases of PRC2 have been associated with the prevention of pluripotency during cell differentiation, while PRC2 activity blocks hormone-mediated SE [98]. Compounds that inhibit enzymatic activities responsible of these epigenetic marks have been used in several in vitro embryogenesis systems, to manipulate ubiquitous epigenetic changes for promoting SE. Some epigenetic modulators that have been shown to promote SE induction are the inhibitors of DNA methyltransferases azacytidine and zeburaline [99,100], the inhibitor of histone methyltransferase, specific for H3K9, BIX-01294 [95], and the inhibitors of histone deacetylases trichostatin A or suberoylanilide hydroxamic acid [101,102]. Supplementing the culture medium with these small molecules induces widespread epigenetic changes that produce higher rates of initiation of SE. However, these epigenetic inhibitors also impaired embryo maturation. This effect can be explained by the fact that SE progression and embryo development are characterized by epigenetic features of cell differentiation, particularly by DNA hypermethylation and increasing H3K9me2 [94,95,100,103]. These findings reveal the crucial role of the epigenetic reprogramming in SE induction. Moreover, these studies are opening new possibilities to improve the efficiency of in vitro embryogenesis by the use of epigenetic modulators, which could extend the application of SE into propagation, breeding and conservation programs.

Investigation during recent years has suggested that cell reprogramming, totipotency acquisition and expression during SE is regulated by a complex interacting network, that includes crosstalk of epigenetic marks, transcription factors and auxin, a network that is repressed in somatic cells, but can be activated by exogenous stimuli, like stress or exogenous 2,4-D. In Arabidopsis, it has been proposed that the induction of SE leads to the removal of epigenetic repressor marks as DNA methylation, H3K9me2 or H3K27me3, and to increase histone acetylation, permitting the expression of specific transcription factors, such as LEC1, LEC2, BBM or AGL15, which would activate auxin biosynthesis and signaling, finally leading to totipotency acquisition and SE initiation [83,104]. This proposed SE regulatory network also involves the direct and indirect interactions between transcription factors and auxin homeostasis pathways and regulatory feedback loops [86]. However, much less is known on the regulatory mechanisms of SE in species other than Arabidopsis, and future studies will be necessary to determine the signaling pathways involved in crop and forest species, where SE is routinely developed, and to gain knowledge for the efficient manipulation and application of SE in recalcitrant species of agronomic and environmental interest.

## 5. Concluding Remarks

Although it was assumed that all plant cells are totipotent, recent studies suggest that only some of them remain in a pluripotent state throughout the plant life cycle, and it is from these cells that new organs develop in response to hormonal induction [105]. In addition, self-organizing auxin and CK interactions reset cell identities after wounding, and provide new positional cues for the re-establishment of the missing tissue through the re-deployment of embryonic development pathways [40]. During cell fate reprogramming in mammalian fibroblasts, the OSK (Oct4, Sox2 and Klf4) transcription factors act as pioneer factors to unwrap condensed chromatin and to induce pluripotent stem cell formation [106]. We propose that ARF7 might act as a pioneer transcription factor during hormone-induced callus formation, through their direct interaction with histone methylation modifiers [16]. The maintenance of the pluripotent state of animal stem cells requires hypoxic conditions, whereas higher oxygen tension promotes cell differentiation [107]. Transcriptional responses to hypoxia in Arabidopsis are mainly controlled by a group of five ERF-VII transcription factors, whose abundance is linked to oxygen levels [108,109]. A link between the establishment of hypoxic niches and plant stem function was recently established in the shoot apical meristem [110]. Additionally, in the LR primordia, the ERF-VII transcription factors bind to the promoters of the auxin-induced genes *LBD16* and *LBD18*, and repress their expression [111]. Hence, low oxygen levels within the new LR primordium might directly interfere with auxin signaling, and could contribute to hindering the auxin-induced activation of neighboring pericycle cells, thus allowing a proper spacing between LRs [111]. As new hypoxia-responsive markers are now available [110], the contribution of low oxygen availability to hormone-induced callus formation could now be elucidated (Figure 1a).

Wounding also promotes tissue regeneration through an orchestrated network of AP2/ERF transcription factors that drive local auxin biosynthesis. Stress conditions can induce somatic cell reprogramming and totipotency expression in a number of cell types, through epigenetic regulators, a complex network of TFs and auxin homeostasis genes that promote embryo formation and plant regeneration (Table 1). Cell trafficking of transcription factors, the establishment of hypoxic niches and step-wise epigenetic reprogramming of regeneration-competent cells are emerging regulators of the tissue regeneration process, and further experiments using single-cell RNA sequencing, marker analysis and protein-protein and protein-DNA complex purification will enhance our understanding in this fascinating research field.

## Figures and Tables

**Table 1:** Some key regulatory factors (transcription factors, epigenetic regulators and others involved in auxin homeostasis) involved in plant regeneration.

Genes	Abbreviations	Function in Plant Regeneration	Molecular Function
*ABA INSENSITIVE 3*	*ABI3*	Quantitatively regulates BBM-mediated somatic embryogenesis. Acts as a positive regulator	Dof-type transcription factor
*ABERRANT LATERAL ROOT FORMATION 4*	*ALF4*	Formative divisions of XPP cells during LR formation. Callus formation upon CIM induction	SCF^TIR1^ regulation
*AGAMOUS LIKE 15*	*AGL15*	Activates auxin biosynthesis, leading to totipotency acquisition and SE initiation	MADS domain transcription factor
*ANTHRANILATE SYNTHASE α1*	*ASA1*	Tryptophan biosynthesis	Oxo-acid-lyase enzyme
*ARABIDOPSIS RESPONSE REGULATOR 1* and *12*	*ARR1 and 12*	Involved in CK-mediated ESR1 induction in order to promote shoot regeneration	Type-B Arabidopsis response regulator transcription factors
*ARABIDOPSIS TRITHORAX-RELATED 2*	*ATXR2*	Positively regulates *LBD16* and *LBD29* expression upon CIM induction	Histone lysine methyltransferase
*AUXIN RESISTANT 3*	*AXR3, IAA17*	Transcriptional repressor upon low auxin levels. Controls stem cell maintenance	Aux/IAA corepressor
*AUXIN RESPONSE FACTOR 7* and *19*	*ARF7 and 19*	LR formation / Positively regulates LBD16 and *LBD29* expression upon CIM induction	Auxin-responsive transcription factor
*BABY BOOM*	*BBM, PLT4, AIL5*	Its ectopic expression can also directly reprogram somatic cells and induce SE in the absence of exogenous stimuli	AP2/ERF transcription factor
*BASIC REGION/LEUCINE ZIPPER MOTIF 59*	*bZIP59*	Interacts with LBD16 upon CIM induction	bZIP transcription factor
*E2 PROMOTER BINDING FACTOR a*	*E2Fa*	DNA replication	E2F transcription factor
*ENHANCER OF SHOOT REGENERATION 1*	*ESR1*	Induces the expression of key shoot regulators (*CUC1*, *RAP2.6L*, *ESR2*, *WUS*, and *STM*) to promote shoot regeneration	AP2/ERF transcription factor
*ETHYLENE RESPONSE FACTOR 109*	*ERF109*	Up-regulates *ERF115* expression. Up-regulates ASA1 expression, probably involved in the auxin biosynthetic pathway	AP2/ERF transcription factor
*ETHYLENE RESPONSE FACTOR 115*	*ERF115*	Acts as as a rate-limiting factor for quiescent center (QC) cell division after DNA damaging stress. Involved in *WIND1* up-regulation upon wound signaling	AP2/ERF transcription factor
*FUSCA 3*	*FUS3*	Involved in embryo development. Essential for successful SE	B3 domain-containing transcription factor
*GENERAL CONTROL NONREPRESSED 5*	*GCN5*, *HAG1*	Root stem cell niche maintenance. Callus pluripotency and shoot induction upon SIM	Histone acetyltransferase
*JASMONATE-ZIM DOMAIN PROTEINS*	*JAZ PROTEINS*	Represses de novo root formation in Arabidopsis leaf explants. Their destabilization allows the action of positive regulators	Jasmonate zinc-finger inflorescence meristem domain transcription factor
*JUMONJI C DOMAIN-CONTAINING 30*	*JMJ30*, *JMJD5*	Positively regulates *LBD16* and *LBD29* expression upon CIM induction	Histone lysine demethylase
*LATERAL ORGAN BOUNDARIES DOMAIN 16, 17, 18* and *29*	*LBD16, 17, 18 and 29*	Callus formation upon CIM induction	LOB-domain transcription factor
*LEAFY COTYLEDON 1*	*LEC1*	Its ectopic expression can also directly reprogram somatic cells and induce SE in the absence of exogenous stimuli	B3 domain-containing transcription factor
*LEAFY COTYLEDON 2*	*LEC2*	Its ectopic expression can also directly reprogram somatic cells and induce SE in the absence of exogenous stimuli	B3 domain-containing transcription factor
*LYSINE-SPECIFIC DEMETHYLASE 1-LIKE 3*	*LDL3*	Presumably removes H3K4me2 during callus formation. It may allow the genes for shoot initiation to be expressed after SIM treatment	Histone lysine demethylase
*microRNA156*	*miRNA156*	Reduces *SPL2*, *10* and *11* expression, promoting AR formation	microRNA molecule
*MONOPTEROS*	*MP*, *ARF5*	Hypophysis specification during embryogenesis	Auxin-responsive transcription factor
*MYB94* and *96*	*MYB94 and 96*	Regulates *LBD29* expression upon CIM induction	MYB transcription factors
MYC2	*MYC2*	Acts upstream of *ERF109* as a positive regulator	bHLH transcription factor
*PHYTOCHROME A SIGNAL TRANSDUCTION 1*	*PAT1*	Acts as a partner of ERF115 and induces *WIND1* expression	GRAS transcription factor
*PIN-FORMED 1*	*PIN1*	Auxin transport	Auxin efflux facilitator
*PLETHORA 3, 5* and *7*	*PLT3, 5* and *7*	Induce the expression of genes involved in regeneration competence acquisition (*PLT2*) and differentiation factors (i.e., *CUC2*)	AP2/ERF transcription factor
*POLYCOMB REPRESSIVE COMPLEX 2*	*PRC2*	Di- and tri-methylation of Lys27 on histone H3. PRC2 activity blocks hormone-mediated SE	Histone lysine methyltransferase
*RWP-RK DOMAIN-CONTAINING 4*	*RKD4*, *GRD*	Induces early embryo-specific genes when overexpressed in seedlings. Its ectopic expression can also directly reprogram somatic cells and induce SE in the absence of exogenous stimuli	RWP-RK-type transcription factor
*SOLITARY ROOT 1*	*SLR1*, *IAA14*	Formative divisions of XPP cells during LR formation	Aux/IAA corepressor
*SQUAMOSA PROMOTER BINDING PROTEIN-LIKE 2, 10* and *11*	*SPL2, 10* and *11*	Their up-regulation is linked to a decrease in wound-induced ARs, presumably due to the repression of ABR1, ERF109, ERF115 and RAP2.6L, among others	SPL transcription factor
*TAA-RELATED 2*	*TAR2*	Auxin biosynthesis	Tryptophan aminotransferase enzyme
*TRYPTOPHAN AMINOTRANSFERASE OF ARABIDOPSIS 1*	*TAA1*	Auxin biosynthesis	Tryptophan aminotransferase enzyme
*WOUND INDUCED DEDIFFERENTIATION 1*	*WIND1*, *RAP2.4*	Establishes and maintains dedifferentiated cell status	AP2/ERF transcription factor
*WUSCHEL*	*WUS*	Shoot induction upon SIM	Homeobox transcription factor
*WUSCHEL RELATED HOMEOBOX 11* and *12*	*WOX11 and 12*	Positively regulates *LBD16* and *LBD29* expression upon CIM induction	Homeobox transcription factor
*YUCCA 1 and 4*	*YUC1 and 4*	Auxin biosynthesis	Flavin-containing monooxygenase enzymes
